# Effects of Feed Removal during Acute Heat Stress on the Cytokine Response and Short-Term Growth Performance in Finishing Pigs

**DOI:** 10.3390/ani11010205

**Published:** 2021-01-15

**Authors:** Kouassi R. Kpodo, Alan W. Duttlinger, Jacob M. Maskal, Betty R. McConn, Jay S. Johnson

**Affiliations:** 1 Department of Animal Sciences, Purdue University, West Lafayette, IN 47907, USA; kkpodo@purdue.edu (K.R.K.); aduttlin@purdue.edu (A.W.D.); jmaskal@purdue.edu (J.M.M.); 2 Oak Ridge Institute for Science and Education, Oak Ridge, TN 37830, USA; mcconn@purdue.edu; 3 Livestock Behavior Research Unit, United States Department of Agriculture—Agricultural Research Service, West Lafayette, IN 47907, USA

**Keywords:** acute heat stress, cytokine, feed removal, growth, inflammation, pigs

## Abstract

**Simple Summary:**

Extreme heat events put pigs at a greater risk for acute heat stress (HS) and increase intestinal permeability and bacterial translocation, resulting in greater inflammatory cytokine production and, subsequently, reduced growth performance. To combat the negative effects of HS, a quick return of body temperature to euthermia is necessary. However, the return of body temperature to euthermia can be delayed by feed access during recovery, which may have detrimental effects on intestinal health and future growth performance in pigs. Therefore, the study objective was to determine whether feed removal during and after an acute HS event would improve short-term growth performance and reduce circulating cytokines in finishing pigs. We hypothesized that feed removal during an acute HS event would result in a faster return of body temperature to euthermia, reduce the cytokine response, and improve short-term growth performance in finishing pigs. Although body temperature and some cytokines were altered by the HS and feeding treatments, overall, few beneficial effects of feed removal during acute HS were detected in finishing pigs in the present study.

**Abstract:**

The study objective was to evaluate the effects of feed removal during acute heat stress (HS) on the cytokine response and its short-term effect on growth performance in finishing pigs. Thirty-two pigs (93.29 ± 3.14 kg initial body weight; 50% barrows and 50% gilts) were subjected to thermoneutral (TN; 23.47 ± 0.10 °C; *n* = 16 pigs) or HS (cycling of 25 to 36 °C; *n* = 16 pigs) conditions for 24 h. Within each temperature treatment, 50% of the pigs were provided with feed (AF; *n* = 8 pigs/temperature treatment) and 50% of the pigs had no feed access (NF; *n* = 8 pigs/temperature treatment). Following the 24 h temperature and feeding treatment (TF) period, all pigs had ad libitum access to feed and water and were maintained under TN conditions for 6 d. During the first 12 h of the TF period, gastrointestinal (T_GI_) and skin (T_sk_) temperatures were recorded every 30 min. Serum cytokines were determined at 0, 4, 8, 12, and 24 h during the TF period and on Days 3 and 6 of the post-TF period. Average daily gain (ADG) and average daily feed intake were measured on Days 1, 3, and 6 of the post-TF period. Behavioral data were collected from Days 1 to 6 of the post-TF period. Heat stress increased (*p* < 0.02) the T_GI_ and T_sk_. During the post-TF period, interleukin-1α was greater (*p* < 0.01) in HS + NF compared to HS + AF and TN + NF pigs. From Days 1 to 2 of the post-TF period, the ADG was reduced (*p* < 0.01) in TN + AF compared to HS + AF, HS + NF, and TN + NF pigs. In conclusion, feed removal during an acute HS challenge did not reduce the cytokine response or improve short-term growth performance in finishing pigs.

## 1. Introduction

Elevated environmental temperatures subject pigs to heat stress (HS) and cause substantial economic losses to the U.S. swine industry [1] due to reduced performance and carcass quality as well as increased morbidity and mortality (as reviewed by [2]). Heat stress causes a well-described increase in intestinal permeability, resulting in greater bacterial translocation and increased systemic inflammation [3], which persists even after the HS insult has ceased [4,5]. In addition, HS causes inflammatory cytokines to be released into circulation from muscles and negatively impacts animal health [6]. These harmful effects of HS are expected to increase as global temperatures continue to rise, and extreme heat events become more frequent [7]. Extreme heat events during summer months put pigs at a greater risk for acute hyperthermia due to their limited ability to use evaporative heat loss and greater metabolic rates [8]. Therefore, identifying risk factors and mitigating the negative effects of acute hyperthermia are of utmost importance for maintaining producer profitability and improving pig health and welfare.

When exposed to elevated environmental temperatures, pigs reduce feed intake to decrease metabolic heat load [8]. However, feed intake increases immediately after the heat load is removed [9], and this has the potential to add heat to the body and delay the return to euthermia due to the heat from nutrient processing [10]. Previous studies in pigs given feed access compared with those not given feed access during HS recovery have demonstrated that the body temperature return to euthermia is delayed [11], and this may exacerbate the cytokine response since the negative consequences of HS may be related to the intensity and duration of heat exposure and subsequent body temperature increase [12]. Therefore, the study objective was to evaluate the effects of feed removal during an acute heat event on the cytokine response and the short-term effects on growth performance in finishing pigs. We hypothesized that feed removal during an acute heat event would result in a more rapid return of body temperature to euthermia, reduce the cytokine response, and improve short-term growth performance in finishing pigs.

## 2. Materials and Methods

### 2.1. Animal and Experimental Design

All procedures involving animals were approved by the Purdue University Animal Care and Use Committee (PACUC no. 1811001826), and animal care and use followed the *Guide for the Care and Use of Agricultural Animals in Research and Teaching* [13]. The study was conducted in West Lafayette, IN, USA, during February 2019. In two repetitions, 32 crossbred (Duroc x (Landrace x Yorkshire)) pigs (*n* = 16 barrows and 16 gilts; 93.29 ± 3.14 kg initial body weight (BW)) were used. One day prior to the experiment, all the pigs were moved into individual pens (1.22 × 2.01 m) within two environmental rooms (thermoneutral (TN) and HS) at the Purdue University Animal Sciences Research and Education Center. Within each environmental room, two data loggers (HOBO data loggers for temperature/relative humidity (RH); manufacturer calibrated accuracy, ± 0.2 °C; Onset, Bourne, MA) were used to record the ambient temperature (T_A_) and relative humidity (RH) at 5 min intervals for the duration of the experiment. At 1500 h on the same day, the pigs were moved into the environmental rooms; each pig was orally administered a CorTemp temperature sensor (model, HT150002; manufacturer calibrated accuracy, ± 0.1 °C; HQ, Inc, Palmetto, FL) to monitor gastrointestinal temperature (T_GI_). The temperature sensor was expected to be located between the duodenum and the jejunum on the following day when treatments were applied as previously determined in similarly sized pigs [5].

**Figure 1:**
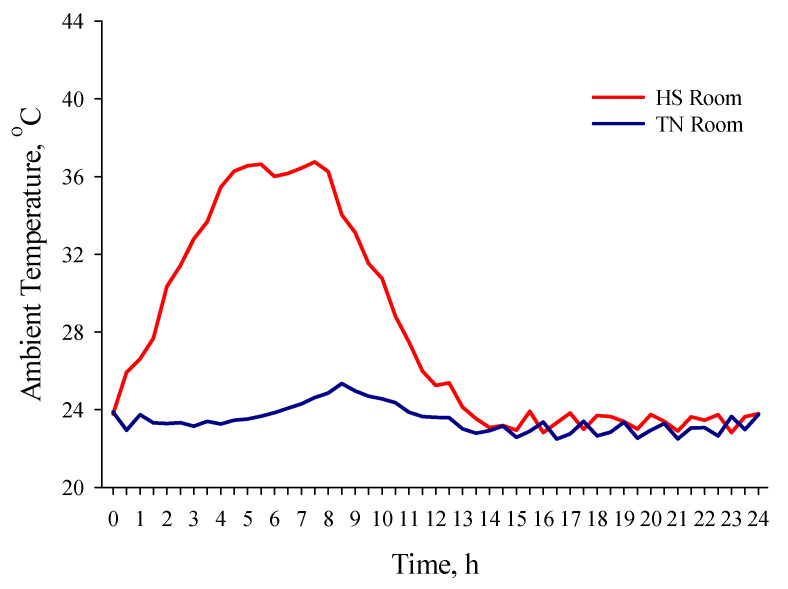
Ambient temperature by time during the 24 h of cyclic heat stress in thermoneutral (TN) and heat stress (HS) rooms.

For the experiment, pigs were subjected to either TN conditions (*n* = 16 pigs; 23.47 ± 0.10 °C; 62.49 ± 0.61% RH) or cyclic HS conditions (*n* = 16 pigs) for 24 h. To achieve the cyclic HS, T_A_ was gradually increased from 23.78 to 36.39 °C over a 4 h period, and then maintained constant at 36.39 ± 0.09 °C for another 4 h. Thereafter, T_A_ was gradually decreased to 25.26 ± 0.11 °C, over a 4 h period, where it remained for the next 12 h (Figure 1). During this 24 h period, within each temperature treatment, one half of the pigs had ad libitum feed access (AF; *n* = 8 pigs/temperature treatment), whereas the other half did not have feed access (NF; *n* = 8 pigs/temperature treatment); however, all the pigs had ad libitum access to water. Following the 24 h period of temperature and feeding treatments, which is hereafter referred to as the TF period, all the pigs were kept under TN conditions (23.30 ± 0.81 °C; 57.22 ± 10.48%) as defined by the *Guide for the Care and Use of Agricultural Animals in Research and Teaching* [13] for 6 d, and a 12L:12D light cycle starting at 0800 h was maintained. All the pigs had ad libitum feed and water access for the remainder of the trial, and they were fed a standard corn and soybean meal diet formulated to meet or exceed nutrient requirements for finishing pigs [14].

### 2.2. Body Temperature Indices

The T_GI_ was measured at 30 min intervals in all the pigs for the first 12 h of the TF period using the pre-administered CorTemp temperature sensors. The skin temperature (T_Sk_) was measured in all the pigs at 30 min intervals for the first 12 h of the TF period by taking a broad side photo of each pig using an infrared camera (FLIR Model T440; accuracy, ±0.1 °C; emissivity = 0.98; FLIR Systems Inc., Wilsonville, OR, USA). The photos were analyzed with the FLIR Tools Software (Version 5.13) by one individual blind to the treatments. The T_Sk_ was determined by drawing a standardized circle on the trunk area (all skin caudal to the neck and dorsal to the elbow and stifle) and recording the mean temperature.

### 2.3. Blood Collection and Analyses

One 10 mL blood tube (BD vacutainers; Franklin Lakes, NJ; serum) was collected from all the pigs via jugular venipuncture. Blood was collected at 0, 4, 8, 12, and 24 h during the 24 h TF period, and on Days 2 and 6 at 0800 h during the post-TF period. Blood samples were centrifuged at 1900× *g* for 15 min at 4 °C to collect serum. Serum samples were stored at −80 °C and later submitted to the University of Minnesota Cytokine Reference Laboratory for cytokine (interleukin (IL)-1α, IL-1β, IL-6, IL-12, IL-10, and tumor necrosis factor α (TNFα)) analyses using a multiplex assay. The intra-assay and inter-assay coefficients of variation for the cytokines were less than 10% and 20%, respectively. The TNFα concentrations were below the detectable limits and were not considered for further analysis.

### 2.4. Production Parameters

Body weight was measured initially on Day 0 and at the end of the 24 h TF period. Feed intake and body weight were measured on Days 2 and 6 after the TF period. Body weight gain during the TF period, and average daily feed intake (ADFI) and average daily body weight gain (ADG) from Days 1 to 2, Days 2 to 6, and Days 1 to 6 of the post-TF period were calculated and used in the growth performance data analyses.

### 2.5. Behavior Recording and Analyses

The pigs were video-recorded during 12 h of light and 12 h of dark from Days 1 to 6 of the post-TF period using ceiling-mounted cameras (Panasonic WV-CP254H, Matsushita Electric Industrial Co. Ltd., Osaka, Japan). Each camera was oriented to capture two adjacent pens (1 AF and 1 NF pig). The video was analyzed in Observer XT 11.5 (Noldus; Wageningen, The Netherlands) using the scan-sampling technique at 10 min intervals for consumption behavior and posture by two trained individuals who were blind to the treatments and maintained at least 90% agreement. Consumption behavior included feeding, drinking, and other. Posture included sitting, lying, and standing. A definition of each behavior is presented in an ethogram (Table 1). Consumption and posture data were determined for 24 h (0800–0800 h) and further separated by daytime (0800–2000 h) and nighttime (2000–0800 h) for analysis.

### 2.6. Statistics

Data were analyzed in a 2 × 2 factorial treatment arrangement (temperature treatment (TN and HS) and feeding treatment (AF and NF)) using the PROC MIXED procedure in SAS 9.4 (SAS Institute Inc., Cary, NC). The linear additive model was Y_ijk_ = µ + T_i_ + F_j_ + K_k_ + T × F_ij_ + e_ijk,_ where Y = the dependent variable of interest, µ = the mean, T = the temperature treatment, F = the feeding treatment, K = the replication, and e = the error term. The cytokine data were analyzed with repeated measures using the hour within the TF period (4, 8, 12, and 24 h) or day post-TF period (2 and 6) as the repeated effect when required. For cytokine analyses, the initial concentrations of each variable were used as a covariate when significant, and plate was included in the model as a random factor. Initial BW was included in the growth performance data analyses as a covariate when significant. Growth performance data were analyzed within the TF period and the post-TF period. Within the TF period, 24 h feed intake data were analyzed only for the treatment groups that received feed (TN + AF; HS + AF). For the post-TF period, growth performance data were analyzed separately from Days 1 to 2, Days 2 to 6, and Days 1 to 6. Individual pigs were considered the experimental units, and repetition was included as a random factor in all the analyses. For the behavior data, only 5 d (Days 1, 2, 4, 5, and 6) were analyzed because the pigs’ behavior was disturbed by the weighing and blood collection on Day 3. A percent of total observations for each pig was calculated and used in the analyses. Consumption behavior (feeding % and drinking %) and posture (standing %, sitting %, and lying %) data were determined for 24 h (0800–0800 h) and further separated by daytime (0800–2000 h) and nighttime (2000–0800 h) for analysis. Other behavior was not included in the final analysis. The behavior and cytokine data were log-transformed to meet normality assumptions when needed, and back-transformed least-squares means are reported. Hour, day, and sex were included as fixed effects in each analysis; however, they are reported only when significant, for clarity. Statistical significance was considered at *p* ≤ 0.05, and a tendency was defined as 0.05 < *p* ≤ 0.10.

## 3. Results

### 3.1. Body Temperature

#### 3.1.1. Gastrointestinal Temperature

**Figure 2:**
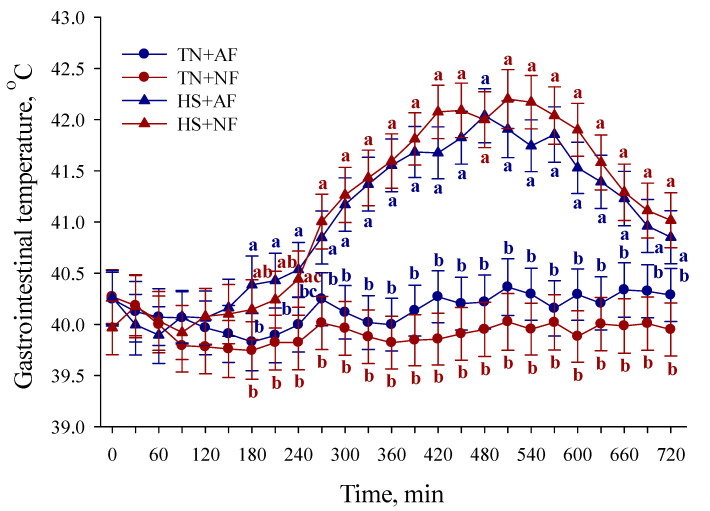
Effects of feed removal on gastrointestinal temperature recorded every 30 min in finishing pigs during the first 12 h of temperature and feeding treatment period. TN: thermoneutral; HS: heat stress; AF: access to feed; NF: no access to feed. Error bars indicate ± 1 SEM. Letters a, b, and c indicate differences at each 30 min time point (*p* < 0.01).

During the TF period, the T_GI_ was greater overall (*p* < 0.01; 1.08 °C) in HS compared to TN pigs, regardless of the feeding treatment (Figure 2). At 180 and 210 min of the TF period, the T_GI_ was greater (*p* < 0.01; 0.59 °C) in HS + AF compared to TN + AF and TN + NF pigs (Figure 2). At 240 min of the TF period, the T_GI_ was greater (*p* < 0.01; 0.62 °C) in HS + AF compared to TN + AF and TN + NF pigs, whereas the T_GI_ was greater (*p* < 0.01; 0.62 °C) in HS + NF compared to TN + NF pigs (Figure 2). From 270 to 720 min of the TF period, the T_GI_ was greater (*p* < 0.01; 1.49 °C) in HS compared to TN pigs, regardless of the feeding treatment (Figure 2). No other T_GI_ differences were detected (*p* > 0.10) during the TF period (Figure 2).

#### 3.1.2. Skin Temperature

**Figure 3:**
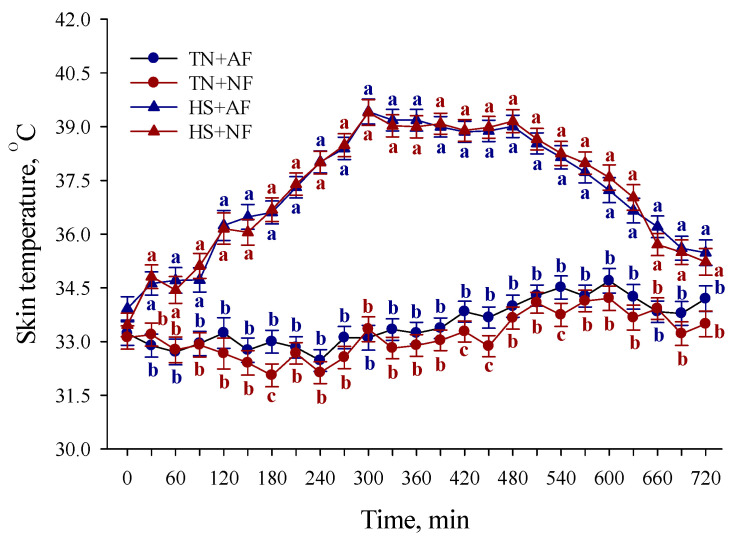
Effects of feed removal on skin temperature recorded every 30 min in finishing pigs during the first 12 h of temperature and feeding treatment period. TN: thermoneutral; HS: heat stress; AF: access to feed; NF: no access to feed. Error bars indicate ± 1 SEM. Letters a, b, and c indicate differences at each 30 min time point (*p* < 0.01).

During the TF period, the T_sk_ was greater overall (*p* < 0.01; 4.02 °C) in HS compared to TN pigs, regardless of the feeding treatment (Figure 3). At 180, 420, 450, and 540 min of the TF period, the T_sk_ was greater (*p* < 0.01; 0.94, 0.55, 0.80, and 0.76 °C, respectively) in TN + AF compared to TN + NF pigs (Figure 3). No other T_sk_ differences were detected (*p* ≥ 0.19) during the TF period (Figure 3).

### 3.2. Cytokines

#### 3.2.1. TF Period

**Figure 4:**
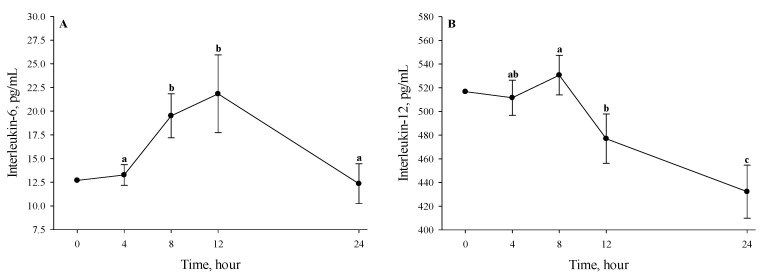
Effects of 24 h temperature and feeding treatment on (**A**) serum interleukin-6 and (**B**) serum interleukin-12 measured every 4 h in finishing pigs. Error bars indicate ± 1 SEM. Letters a and b indicate hourly differences (*p* < 0.01). Hour 0 data are for descriptive purposes only.

During the TF period, serum IL-1α, IL-6, IL-10, and IL-12 were greater overall (*p* ≤ 0.03%; 47.23%, 39.91%, 152.60%, and 24.78%, respectively) in AF compared to NF pigs, regardless of the temperature treatment (Table 2). A time difference was observed, where IL-6 was greater (*p* < 0.01) at 8 h (19.51 ± 2.33 pg/mL) and 12 h (21.83 ± 4.11 pg/mL) compared to 4 h (13.26 ± 1.10 pg/mL) and 24 h (12.34 ± 2.10 pg/mL) of the TF period, regardless of the temperature and feeding treatments (Figure 4A). No differences were detected between 8 and 12 h or 4 and 24 h of the TF period (Figure 4A). Serum IL-12 was greater (*p* < 0.01) at 4 h (511.59 ± 14.76 pg/mL), 8 h (530.67 ± 16.74 pg/mL), and 12 h (477.03 ± 20.78 pg/mL) when compared to 24 h (432.22 ± 22.44 pg/mL) of the TF period, regardless of the temperature and feeding treatments, but no differences were detected between 4 and 8 h or 4 and 12 h of the TF period (Figure 4B). Interleukin-12 was greater overall (*p* = 0.05) in gilts (514.81 ± 18.67 pg/mL) compared to barrows (463.29 ± 18.67 pg/mL), regardless of the temperature and feeding treatments (data not presented). No other cytokine differences were observed (*p* ≥ 0.11) during the TF period (Table 2; Figure 4).

#### 3.2.2. Post-TF Period

On Days 2 and 6 of the post-TF period, serum IL-1α tended to be greater overall (*p* = 0.09; 45.91%) in HS compared to TN pigs, regardless of the feeding treatment (Table 2). Overall, IL-6 and IL-12 were greater (*p* < 0.01; 73.49% and 21.73%, respectively) in AF compared to NF pigs, regardless of the temperature treatment (Table 2). Interleukin-10 tended to be greater overall (*p* = 0.06; 169.71%) in AF compared to NF pigs, regardless of the temperature treatment (Table 2). Interleukin-1α was greater overall (*p* < 0.01; 201.41%) in HS + NF compared to HS + AF and TN + NF pigs (Table 2). In addition, IL-1α was increased overall (*p* < 0.01; 265.37%) in TN + AF compared to TN + NF pigs (Table 2). Interleukin-6 was reduced overall (*p* < 0.01; 75.61%) in TN + NF compared to TN + AF, HS + AF, and HS + NF pigs (Table 2). Interleukin-1β tended to be greater overall (*p* = 0.08; 89.06%) in TN + AF compared to HS + AF pigs (Table 2). Interleukin-12 tended to be greater (*p* = 0.10; 29.77%) in TN + AF pigs compared to TN + NF, HS + AF, and HS + NF pigs (Table 2). No other cytokine differences were detected on Day 2 or 6 of the post-TF period (*p* > 0.17; Table 2).

### 3.3. Growth Performance

#### 3.3.1. TF Period

During the 24 h TF period, weight loss was greater overall (*p* = 0.01; 53.37%) in HS compared to TN pigs, regardless of the feeding treatment (Table 3). Weight loss was greater overall during the TF period (*p* < 0.01; 186.39%) in NF compared to AF pigs, regardless of the temperature treatment (Table 3). Weight loss was reduced (*p* = 0.02; 37.92%) in HS + AF pigs compared to HS + NF and TN + NF pigs (Table 3). In addition, weight loss was increased (*p* = 0.02; 102.6%) in HS + AF, HS + NF, and TN + NF pigs versus TN + AF pigs (Table 3). No other growth performance differences were detected during the TF period (*p* > 0.10; Table 3).

#### 3.3.2. Post-TF Period

##### Average Daily Body Weight Gain

When data were analyzed for Days 1 to 2 of the post-TF period, the ADG was found to be greater overall (*p* < 0.01; 75.93%) in NF compared to AF pigs, regardless of the temperature treatment (Table 3). From Days 1 to 2 of the post-TF period, the ADG was increased overall (*p* < 0.01; 147.86%) in HS + AF, HS + NF, and TN + NF pigs compared to TN + AF pigs (Table 3). In addition, from Days 1 to 2 of the post-TF period, the ADG was reduced overall (*p* < 0.01; 29.76%) in HS + AF compared to TN + NF pigs (Table 3). When data were analyzed for the entire post-TF period (Days 1 to 6), the ADG was greater overall (*p* < 0.01; 40.29%) in NF compared to AF pigs, regardless of the temperature treatment (Table 3). From Days 1 to 6 of the post-TF period, the ADG was increased overall (*p* < 0.01; 32.93%) in TN + NF pigs and was reduced in TN + AF pigs (32.93%) compared to HS + AF and HS + NF pigs (Table 3). In addition, from Days 1 to 6 of the post-TF period, the ADG was greater (*p* < 0.01; 98.18%) in TN + NF pigs compared to TN + AF pigs (Table 3). No other ADG differences were detected during the post-TF period (*p* > 0.10; Table 3).

##### Average Daily Feed Intake

From Days 1 to 2 of the post-TF period, the ADFI was reduced (*p* = 0.02; 12.31%) in HS compared to TN pigs, regardless of the feeding treatment (Table 3). From Days 1 to 2 of the post-TF period, the ADFI was increased (*p* = 0.02; 13.70%) in NF compared to AF pigs, regardless of the temperature treatment (Table 3). From Days 1 to 2 of the post-TF period, the ADFI was greater (*p* = 0.01; 14.42%) for barrows compared to gilts, regardless of the temperature and feeding treatment (Table 4). From Days 2 to 6 of the post-TF period, the ADFI tended to be greater (*p* = 0.07; 10.36%) in TN + NF compared to TN + AF and HS + NF pigs (Table 3). From Days 2 to 6 of the post-TF period, the ADFI was greater overall (*p* = 0.01: 11.40%) in barrows compared to gilts, regardless of the temperature and feeding treatment (Table 4). From Days 2 to 6 of the post-TF period, the ADFI was reduced overall in TN + AF gilts and HS + NF gilts (*p* = 0.01; 18.98% and 19.59%, respectively) when compared to TN + AF barrows, TN + NF barrows, TN + NF gilts, HS + NF barrows, and HS + AF gilts (Table 4). When data were analyzed for the entire post-TF period (Days 1 to 6), the ADFI tended to be decreased overall (*p* = 0.08; 5.84%) in HS compared to TN pigs, regardless of the feeding treatment (Table 3). From Days 1 to 6 of the post-TF period, the ADFI was greater (*p* = 0.03; 13.09%) in TN + NF pigs compared to TN + AF, HS + AF, and HS + NF pigs (Table 3). From Days 1 to 6 of the post-TF period, the ADFI was greater overall (*p* < 0.01; 10. 07%) in barrows compared to gilts (Table 4). From Days 1 to 6 of the post-TF period, the ADFI was reduced (*p* = 0.02) in TN + AF gilts and HS + NF gilts (19.76% and 17.94%, respectively) when compared to TN + AF barrows, HS + NF barrows, TN + NF gilts, and HS + NF barrows (Table 4). No other ADFI differences were detected during the post-TF period (*p* > 0.10; Table 3 and Table 4).

##### Feed Efficiency

From Days 1 to 2 of the post-TF period, the body weight gain-to-feed intake ratio (G:F) was greater overall (*p* < 0.01; 37.27%) in HS compared to TN pigs, regardless of the feeding treatment (Table 3). From Days 1 to 2, the G:F was greater overall (*p* < 0.01; 49.67%) in NF compared to AF pigs, regardless of the temperature treatment (Table 3). From Days 1 to 2, the G:F was increased (*p* < 0.01; 138.29%) in TN + NF, HS + AF, and HS + NF pigs when compared to TN + AF pigs (Table 3). When the data were analyzed for the entire post-TF period (Days 1 to 6), the G:F was greater overall (*p* < 0.01; 32.61%) for NF compared to AF pigs, regardless of the temperature treatment (Table 3). From Days 1 to 6 of the post-TF period, the G:F was increased overall (*p* < 0.01; 59.46%) in TN + NF, HS + AF, and HN + NF pigs when compared to TN + AF pigs (Table 3). No other G:F differences were detected (*p* ≥ 0.12) during the post-TF period (Table 3).

### 3.4. Behavior

#### 3.4.1. Overall (0800–0800 h)

From 0800 to 0800 h, the drinking behavior was greater (*p* = 0.03; 64.86%) in HS compared to TN pigs, regardless of the feeding treatment (Table 5). Standing behavior tended to be reduced (*p* = 0.06; 19.38%) in HS + AF compared to TN + AF pigs from 0800 to 0800 h (Table 5). Sitting behavior tended to be greater (*p* = 0.10; 40.91%) in HS compared to TN pigs, regardless of the feeding treatment from 0800 to 0800 h (Table 5). Lying behavior was increased (*p* = 0.02; 2.93%) in TN + NF and HS + AF pigs when compared to TN + AF pigs from 0800 to 0800 h (Table 5). No other overall behavior differences were detected (*p* ≥ 0.12; Table 5).

#### 3.4.2. Daytime (0800–2000 h)

During the daytime, eating behavior tended to be reduced overall (*p* = 0.06; 26.00%) in HS + AF compared to TN + AF pigs (Table 5). Daytime drinking behavior tended to be increased overall (*p* = 0.07; 38.78%) in NF compared to AF pigs, regardless of the temperature treatment (Table 5). Standing behavior was reduced during the daytime (*p* = 0.01; 29.30%) in HS + AF compared to TN + AF pigs (Table 5). Standing behavior tended to be reduced in the daytime (*p* = 0.07; 13.70%) for HS compared to TN pigs, regardless of the feeding treatment (Table 5). Sitting behavior was greater during the daytime (*p* = 0.05; 59.29%) in HS compared to TN pigs, regardless of the feeding treatment (Table 5). Lying behavior was increased during the daytime (*p* = 0.01; 36.36%) in TN + NF and HS + AF pigs versus TN + AF pigs (Table 5). No other daytime behavior differences were detected (*p* ≥ 0.12; Table 5).

#### 3.4.3. Nighttime (2000–0800 h)

During the nighttime, drinking behavior was reduced (*p* < 0.01; 76.63%) in HS compared to TN pigs (Table 5). Standing behavior was greater during the nighttime (*p* = 0.04) in barrows (7.83 ± 0.71%) compared to gilts (6.23 ± 0.69%; data not presented). No other nighttime behavior differences were detected (*p* ≥ 0.33; Table 5).

## 4. Discussion

The thermic effect of feeding increases body temperature [15] and may delay the return to euthermia in pigs recovering from HS. As such, feed removal during HS and subsequent recovery is associated with a more rapid return of body temperature to euthermia [11,16] and may reduce systemic inflammation in pigs [16]. However, contrary to these reports, no body temperature (T_GI_ and T_sk_) differences were detected between the HS + AF and HS + NF pigs in the present study. Although feed intake was not measured during the first 12 h of the TF period, it is well established that HS-exposed pigs reduce feed intake as a mechanism of decreasing the thermic effects of feeding [17,18]. Therefore, the lack of body temperature differences may have been a result of an overall reduction in feed intake in HS + AF pigs that placed them on a similar nutritional plane to the HS + NF pigs and reduced the potential for feeding-induced differences in body temperature. Furthermore, the reasons why the results of the current study contradict previous reports [11,16] may be related to the cooling procedure used or the severity of the heat load imposed. In the previous studies [11,16], pigs were rapidly cooled by cold water dousing following a constant and severe HS. However, in the current study, pigs were exposed to cyclic HS to replicate a natural summer production environment and were not rapidly cooled. Therefore, the lack of feeding by temperature differences during the TF period in the present study suggest that feed removal alone may not be an effective method for promoting a rapid return of body temperature to euthermia following acute HS events.

Heat stress results in tissue damage and subsequent systemic inflammatory responses modulated by pro- and anti-inflammatory cytokines [19,20]. Adaptive mechanisms to maintain euthermia under HS conditions, including reduced visceral perfusion, cause ischemia and increase intestinal endotoxin leakage, which stimulates inflammatory cytokine production [20]. In addition, direct HS effects on skeletal muscle result in cytokine production and leakage into systemic circulation [20,21]. However, in the current study, no pro-inflammatory (IL-1, IL-6, and IL-12) or anti-inflammatory (IL-10) cytokine differences were detected between HS + AF and HS + NF pigs during the TF period. The lack of cytokine differences may have been due to the aforementioned absence of body temperature differences observed between the HS + AF and HS + NF pigs during the first 12 h of the TF period, indicating similar levels of hyperthermia.. In addition to the lack of cytokine response differences between HS + AF and HS + NF pigs, no cytokine differences were detected between HS and TN pigs during the TF period. The reasons for the overall lack of cytokine differences observed in the current study are unknown. However, it is important to note that the effects of HS on inflammatory cytokine concentrations in pigs are not always consistent from study to study (as reviewed by [22]) and may depend on the experimental protocol. For example, the lack of inflammatory cytokine differences in the present study agrees with previous reports where no IL-1β [5,23] or IL-6 [24,25] differences were detected in HS versus TN pigs. Regarding IL-12, while no differences were observed between HS and TN pigs in the current study, circulating IL-12 was actually decreased by HS according to previous reports [26]. The differences between studies may be due to the HS protocols, whereby some studies utilize cyclic HS to mimic natural conditions [27,28] and others use either constant HS [26] or severe and acute HS [5]. In addition, blood collection processes may play a role in detecting circulating cytokines, as blood collection timing and processing can impact the detection of certain cytokines (as reviewed by [29]). Future studies may be needed to determine the effects of a more natural cyclic HS challenge on circulating inflammatory cytokines in pigs relative to either constant HS or acute HS exposure and if blood collection timing influences the presence of these inflammatory markers.

Inflammatory cytokine concentrations were reduced overall in NF compared to AF pigs, regardless of the temperature treatment. Limited data exist on the effects of feeding on circulating cytokine concentrations in pigs; however, studies in humans have reported decreased IL-6 [30,31] and IL-1α [31] during fasting. While the reasons why cytokine concentrations were reduced in NF compared to AF pigs in the current study are unclear, it is possible that the reduced energy availability for NF pigs may have inhibited their immune response since the immune system requires glucose for activation and function [32]. As a result, the production of cytokines by immune cells (monocytes, macrophages, etc.) may have been decreased (as reviewed by [33]) due to reduced energy availability. However, further research is needed to confirm this hypothesis and better understand the effects of fasting and feeding on circulating inflammatory cytokines in pigs.

Although no cytokine differences were detected in the TF period, IL-1α was greater overall in HS + NF compared to HS + AF pigs during the post-TF period. Interleukin-1α is a pro-inflammatory cytokine known to regulate the body’s response to infection and inflammation [34]. It is increased by hyperthermia as evidenced by elevated concentrations in heat stroke victims before and after cooling [35]. It is possible that the increased IL-1α in HS + NF pigs was due to re-feeding, as IL-1α was reduced overall in NF compared to AF pigs during the TF period. Alternatively, re-feeding following a 24 h period of fasting may have caused intestinal damage as previously reported [36], and this may have increased IL-1α because intestinal damage is associated with increased circulating cytokine production [5,37]. However, these hypotheses require further investigation.

During HS, growth performance is reduced, and this decrease can depend on HS intensity and duration [17]. In the current study, the 24 h TF period resulted in a marked decrease in BW for HS compared to TN pigs, regardless of the feeding treatment. These data agree with previous reports [25,38] and may be partially related to gut filling because HS reduces feed intake [17,39,40]. However, no feed intake differences were detected between HS + AF and TN + AF pigs during the TF period, and the numerical difference in feed intake did not match the BW loss difference. While a lack of feed intake differences during the TF period is somewhat surprising considering the greater BW loss for HS + AF versus TN + AF pigs, it corresponds with a previous report where BW loss was greater in pigs exposed to a 12 h cyclical HS challenge when compared to TN controls, independent of feed intake differences [41]. Furthermore, the lack of feed intake differences may be due to the cyclical nature of the HS challenge, whereby HS pigs may have compensated for the decreased feed intake during the hotter daytime period by consuming more feed in the nighttime hours when it was cooler. Therefore, the difference in BW loss for HS + AF compared to TN + AF pigs during the TF period is likely due to other factors such as water loss due to dehydration.

In addition to the effects of HS, BW loss during the TF period was greater overall in NF compared to AF pigs. However, this was expected because NF pigs did not have feed access and, thus, gut filling would have been reduced. Furthermore, the ADG and G:F were greater in HS + AF, HS + NF, and TN + NF compared to TN + AF pigs from Days 1 to 2 of the post-TF period. The increased ADG from Days 1 to 2 was likely due to the effects of greater gut filling caused by the increasing feed intake and hydration following HS exposure and/or feed restriction. As a result, increased gut filling due to re-feeding may also explain why the G:F was greater than 1.00 during this time frame for the TN + NF, HS + AF, and HS + NF treatment groups, since BW gain over that of feed intake is not generally possible in finishing pigs. Furthermore, the increased ADG and G:F during this time frame may explain the overall Day 1 to 6 ADG and G:F increase for TN + NF, HS + AF, and HS + NF versus TN + AF pigs. Additionally, the ADFI was greater in TN + AF and HS + NF barrows compared to TN + AF and HS + NF gilts. While this is consistent with previous literature indicating that barrows have greater feed intake than gilts [42,43], it is unclear why no differences were observed between TN + NF barrows and gilts or HS + AF barrows and gilts. Therefore, further research should be conducted to clarify the effects of sex, feeding, and HS on the ADFI in pigs.

In addition to reducing feed intake, HS altered behavioral measures in pigs. The pigs exposed to HS had an increased drinking frequency relative to the TN pigs during the post-TF period, regardless of the feeding treatment. The increased drinking frequency was expected, has been previously observed in HS pigs [44], and is likely due to HS-induced reductions in body water reserves due to greater evaporative heat loss [45]. In addition, the HS pigs had an increase in sitting behavior during the daytime compared to the TN-exposed pigs. Previous reports have associated a greater sitting frequency with increased stress in pigs following transport under HS conditions [46] and in barren environments [47]. Taken together, the increase in the sitting and drinking behavior for the HS-exposed pigs may indicate that the hyperthermia-induced stress response persisted even after the temperature insult ceased.

Although no recovery treatment and feeding treatment interaction was detected for T_GI_ in the current study, some caveats should be noted. It was previously determined that when the CorTemp sensor was orally administered by 1500 h on the day prior to temperature challenge, the sensor would be located between the duodenum and the jejunum during the first 12 h of the TF period on the following day [4]. However, this was established in pigs with access to feed. In the current study, one half of the pigs were fasted, and the emptiness of the intestine may have affected the CorTemp sensor movement. Therefore, it is important to determine how fasting might affect the sensor movement for future studies. Regardless, these data improve our understanding of fasting effects on the intestinal temperature during HS.

## 5. Conclusions

We hypothesized that feed removal during an acute heat event would result in a faster return of body temperature to euthermia, reduce the cytokine response, and improve short-term growth performance in finishing pigs. However, contrary to our hypothesis, feed removal during an acute heat event did not hasten the return of body temperature to euthermia and reduce the cytokine response in pigs. On the contrary, feed removal appears to have induced a short-term increase in the cytokine response following the TF period, and this may have been a result of re-feeding, which has been shown to increase intestinal damage and can lead to systemic inflammation. In addition, HS increased the sitting frequency during the post-TF period, which may be indicative of a greater stress response. Although this study disproved our hypothesis, it suggests that feed removal alone may not prevent the systemic inflammation mediated by greater cytokine production during HS, and that the negative effects of acute HS on swine welfare and stress responses may last for several days following the initial insult.

## Figures and Tables

**Table 1:** Ethogram used for behavior analyses.

Category	Behavior	Definition
Consumption	Eating	The pig has its head in the feeder
	Drinking	The pig has its snout in contact with the waterer
	Other	Anything other than head in the feeder and snout in contact with the waterer
Posture	Sitting	The pig is on the thigh and forelegs, active or inactive
	Lying	The pig is lying on the floor sternally or laterally
	Standing	The pig is on its four legs, active or inactive

**Table 2:** Serum cytokines measured during the 24 h temperature and feeding treatment (TF) period and the post-TF period (Days 2 and 6) in finishing pigs.

Parameter	Temperature Treatment + Feeding Treatment					*p*-Value		
	TN ^1^ + AF ^2^	TN + NF ^3^	HS ^4^ + AF	HS + NF	SEM	T ^5^	F ^6^	T × F
TF period								
IL^7^-1α, pg/mL	5.59	3.04	5.32	4.37	0.83	0.38	0.02	0.25
IL-1β, pg/mL	16.30	14.32	19.01	9.16	4.27	0.59	0.11	0.26
IL-6, pg/mL	21.36	16.47	17.52	11.32	2.96	0.11	0.05	0.63
IL-10, pg/mL	19.77	7.70	17.69	7.13	5.35	0.82	0.03	0.96
IL-12, pg/mL	560.72	430.67	522.61	437.50	26.50	0.54	<0.01	0.39
Post-TF period								
IL-1α, pg/mL	7.49 ^ab^	2.05 ^c^	4.32 ^bc^	9.60 ^a^	1.59	0.09	0.42	<0.01
IL-1β, pg/mL	24.54 ^x^	13.84 ^xy^	12.98 ^y^	19.79 ^xy^	6.81	0.62	0.78	0.08
IL-6, pg/mL	24.39 ^a^	4.46 ^b^	13.24 ^a^	17.23 ^a^	3.86	0.18	<0.01	<0.01
IL-10, pg/mL	33.73	4.72	22.37	16.08	10.78	0.49	0.06	0.17
IL-12, pg/mL	748.78 ^x^	546.14 ^y^	612.61 ^y^	572.21 ^y^	62.89	0.24	0.01	0.10

^1^ Thermoneutral, ^2^ Access to feed, ^3^ No feed access, ^4^ Heat stress, ^5^ Temperature treatment, ^6^ Feeding treatment, ^7^ Interleukin. ^a,b,c^ letters indicate significant differences (*p* ≤ 0.05) within a row. ^x,y^ letters indicate tendencies (0.05 < *p* ≤ 0.10).

**Table 3:** Growth performance monitored during the 24 h temperature and feeding treatment (TF) period (Day 0) and Days 1 to 2, 2 to 6, and 1 to 6 of the post-TF period in finishing pigs.

Parameter	Temperature Treatment + Feeding Treatment					*p*-Value		
	TN ^1^ + AF ^2^	TN + NF ^3^	HS ^4^ + AF	HS + NF	SEM	T ^5^	F ^6^	T × F
Initial BW ^7^, kg	93.19	93.07	93.10	93.97	1.50	0.73	0.74	0.72
Day 0								
∆BW, kg	−0.40 ^a^	−5.09 ^c^	−3.20 ^b^	−5.22 ^c^	0.53	0.01	<0.01	0.02
Feed intake, kg	2.21	–	1.82	–	0.33	0.41	–	–
Days 1 to 2								
ADG ^8^, kg	1.40 ^c^	4.10 ^a^	2.88 ^b^	3.43 ^ab^	0.28	0.17	<0.01	<0.01
ADFI ^9^, kg	2.98	3.60	2.80	2.97	0.23	0.02	0.02	0.18
G:F ^10^, kg/kg	0.47 ^b^	1.14 ^a^	1.06 ^a^	1.15 ^a^	0.08	<0.01	<0.01	<0.01
Days 2 to 6								
ADG, kg	0.88	0.96	0.91	0.78	0.09	0.38	0.79	0.27
ADFI, kg	2.96 ^y^	3.25 ^x^	3.10 ^xy^	2.93 ^y^	0.12	0.48	0.63	0.07
G:F, kg/kg	0.30	0.29	0.29	0.27	0.03	0.55	0.63	0.69
Days 1 to 6								
ADG, kg	1.10^c^	2.18 ^a^	1.63 ^b^	1.65 ^b^	0.15	0.99	<0.01	<0.01
ADFI, kg	2.97 ^b^	3.37 ^a^	3.02 ^b^	2.95 ^b^	0.10	0.08	0.12	0.03
G:F, kg/kg	0.37 ^b^	0.65 ^a^	0.55 ^a^	0.57 ^a^	0.04	0.24	<0.01	<0.01
Final BW, kg	99.53	100.12	99.84	98.17	0.67	0.33	0.57	0.16

^1^ Thermoneutral, ^2^ Access to feed, ^3^ No feed access, ^4^ Heat stress, ^5^ Temperature treatment, ^6^ Feeding treatment. ^7^ Body weight, ^8^ Average daily body weight gain, ^9^ Average daily feed intake, ^10^ Body weight gain-to-feed intake ratio. ^a,b,c^ letters indicate significant differences (*p* ≤ 0.05) within a row. ^x,y^ letters indicate tendencies (0.05 < *p* ≤ 0.10).

**Table 4:** Effects of temperature and feeding treatments (**TF**) and sex on average daily feed intake, monitored on Days 1 to 2, Days 2 to 6, and Days 1 to 6 of the post-TF period in finishing pigs.

Parameter	Temperature Treatment + Feeding Treatment									*p*-Value			
	TN ^1^ + AF ^2^		TN + NF ^3^		HS ^4^ + AF		HS + NF		SEM	T ^5^	F ^6^	S ^7^	T × F × S
	Barrows	Gilts	Barrows	Gilts	Barrows	Gilts	Barrows	Gilts					
Days 1 to 2													
ADFI ^8^, kg	3.29	2.67	3.74	3.45	3.13	2.47	3.01	2.92	0.28	0.02	0.02	0.02	0.72
Days 2 to 6													
ADFI, kg	3.29 ^a^	2.63 ^b^	3.35 ^a^	3.15 ^a^	3.01 ^ab^	3.19 ^a^	3.25 ^a^	2.61 ^b^	0.17	0.39	0.71	0.01	0.01
Days 1 to 6													
ADFI, kg	3.29 ^a^	2.65 ^b^	3.38 ^a^	3.36 ^a^	3.05 ^ab^	3.00 ^ab^	3.18 ^a^	2.71 ^b^	0.15	0.08	0.12	<0.01	0.02

^1^ Thermoneutral, ^2^ Access to feed, ^3^ No feed access, ^4^ Heat stress, ^5^ Temperature treatment, ^6^ Feeding treatment, ^7^ Sex, ^8^ Average daily feed intake. ^a,b^ letters indicate significant differences (*p* ≤ 0.05) within a row.

**Table 5:** Consumption behavior and posture measured on Days 1, 2, 4, 5, and 6 of post-temperature-and-feeding-treatment period in finishing pigs.

Parameter	Temperature Treatment + Feeding Treatment					*p*-Value		
	TN ^1^ + AF ^2^	TN + NF ^3^	HS ^4^ + AF	HS + NF	SEM	T ^5^	F ^6^	T × F
Consumption								
0800–0800 h								
Eating, %	7.51	6.25	6.18	7.25	1.13	0.82	0.90	0.12
Drinking, %	0.50	0.61	0.91	0.92	0.17	0.03	0.70	0.73
0800–2000 h								
Eating, %	10.50 ^x^	8.11 ^xy^	7.77 ^y^	9.59 ^xy^	1.13	0.60	0.85	0.06
Drinking, %	0.72	0.75	0.75	1.29	0.25	0.78	0.07	0.65
2000–0800 h								
Eating, %	2.95	3.29	3.31	3.45	1.03	0.59	0.61	0.83
Drinking, %	0.30	0.47	0.13	0.05	0.08	<0.01	0.81	0.12
Posture								
0800–0800 h								
Standing, %	13.31 ^x^	11.57 ^xy^	10.73 ^y^	12.19 ^xy^	0.85	0.25	0.87	0.06
Sitting, %	1.02	0.74	1.19	1.29	0.25	0.10	0.47	0.64
Lying, %	84.83 ^b^	87.29 ^a^	87.34 ^a^	85.98 ^ab^	1.23	0.47	0.51	0.02
0800–2000 h								
Standing, %	18.77 ^a^	15.60 ^ab^	13.27 ^b^	16.39 ^ab^	1.28	0.07	0.87	0.01
Sitting, %	1.32	0.94	1.86	1.74	0.35	0.05	0.42	0.62
Lying, %	77.27 ^b^	81.73 ^a^	82.72 ^a^	79.54 ^ab^	1.38	0.23	0.62	0.01
2000–0800 h								
Standing, %	6.98	6.70	7.03	7.41	0.89	0.62	0.95	0.67
Sitting, %	0.35	0.27	0.46	0.28	0.14	0.68	0.33	0.74
Lying, %	92.26	92.75	91.87	91.82	0.90	0.46	0.81	0.76

^1^ Thermoneutral, ^2^ Access to feed, ^3^ No feed access, ^4^ Heat stress, ^5^ Temperature treatment, ^6^ Feeding treatment. ^a,b^ letters indicate significant differences (*p* ≤ 0.05). ^x,y^ letters indicate tendencies (0.05 < *p* ≤ 0.10).

## Data Availability

The data presented in this study are available on request from the corresponding author.
